# Supplementary material for: Predictors of Mortality in Hemodialyzed Patients after SARS-CoV-2 Infection
Source: J Clin Med. 2022 Jan 6;11(2):285. doi: 10.3390/jcm11020285 (PMC8778392; doi:10.3390/jcm11020285)
Supplement: Supplementary file 1 [file jcm-11-00285-s001.zip › jcm-1471480-supplementary.pdf]

Table S1 Predictors of 3 – month mortality. Multivariable logistic regression.

Variable	OR	Lower 95% CI	Upper 95% CI	p-value
Chronic D-vitamin supplementation	0,74	0,24	2,33	0,61
Age > 65 y	2,31	0,45	11,94	0,32
Blood type 0	0,49	0,14	1,70	0,26
D-Dimer > 1500 ng/mL	6,00	1,94	18,53	<0,01
Clinical Frailty Scale >3	8,36	1,81	38,63	<0,01
CRP > 118 mg/L	3,77	1,09	13,02	0,04

Table S2 Baseline characteristic, clinical presentation and laboratory findings on hospital admission of HD patients with COVID-19 who survived and those died during 3 months after discharge.

Variable	Survivors n=80	Died after discharge n=9	p-value
Sex			
Male	41 (51.25)	4 (44.46)	p>0.1
Female	39 (48.75)	5 (55.55)	p>0.1
Age, years	71.0 (61-77)	74.00 (68-82)	p>0.1
Blood group			
A	31(38.0)	5 (55.54)	p>0.1
B	13 (16.25)	2 (22.22)	p>0.1
AB	5 (6.25)	0 (0.0)	p>0.1
0	31 (38.75)	2 (22.22)	p>0.1
Body mass index, kg/m2	26.0 (22.0-29.0)	23.74 (18.53-36.46)	p>0.1
Dialysis vintages, months	37.5 (17-90)	59 (18-88)	p>0.1
Dialysis dose per week, hours	12 (12-12)	12 (12-12)	p>0.1
Past kidney transplantation	7 (8.75)	0 (0.0)	p>0.1
Comorbidities			
Diabetes	42 (52.5)	5 (55.54)	p>0.1
Hypertension	77 (96.25)	9 (100.0)	p>0.1
Chronic pulmonary disease	4 (5.0)	1 (11.11)	p>0.1
Ischemic heart disease	29 (36.25)	4 (44.46)	p>0.1
Congestive heart failure	29 (36.25)	5 (55.54)	p>0.1
Malignancy	6 (7.5)	0 (0.0)	p>0.1
Charlson comorbidity index (age adjusted)	7 (6-9)	8 (7-10)	p>0.1
Clinical Frailty Scale	4 (3-5)	5 (4-6)	p=0.003
Medications:			
ACE inhibitors	15 (18.75)	4 (44.46)	p=0.07
ARBs	8 (10)	1 (11.11)	p>0.1
Calcium channel blockers	25 (31.25)	4 (44.46)	p>0.1
Beta-blockers	59 (73.75)	7 (77.77)	p>0.1
Statins	37 (46.25)	5 (55.54)	p>0.1
Oral anticoagulant	6 (7.5)	1 (11.11)	p>0.1
LMWH between dialysis days	17 (21.25)	3 (33.33)	p>0.1
Active oral vitamin D	57 (71.25)	4 (44.46)	p=0.1
Epoetin beta IU per week	6000 (3500-6000)	6000 (4000-9000)	p>0.1
COVID-19 disease severity on admission			
Asymptomatic-Mild	68 (85)	5 (55.54)	p=0.03
Moderate-Severe	12 (15)	4 (44.46)	p=0.03
Physical examination on admission:			
Heart rate, beats per min	80 (73-90)	85 (81-86.5)	p>0.1
Respiratory rate, breaths per min	16 (15-17)	18 (12-20)	p>0.1
Oxygen saturation, %	95 (92-97)	93 (89.5-95)	p>0.1
Systolic blood pressure, mmHg	130 (122-152)	120 (115-154)	p>0.1
Diastolic blood pressure, mmHg	80 (70-90)	77 (62-80)	p>0.1
Temperature °C	36.7 (36.4-37)	36.7 (36.4-37.4)	p>0.1

Laboratory findings on admission			
White blood cell count, x 10^9/l	5.12 (3.99-8.11)	5.07 (4.2-5.63)	p>0.1
Lymphocyte count/ mm^3	0.92 (0.63-1.4)	0.64 (0.41-0.84)	p>0.1
Hemoglobin, g/dl	11.1 (10.0-11.6)	9.5 (9.1-10.4)	p>0.1
Platelet count, x 10^9/l	195.5 (149-239)	171 (127-205)	p>0.1
CRP, mg/l	34.5 (9.1-95.1)	90.1 (28.5-119)	p>0.01
D-dimer, ng/ml	1106.5 (724.9-1491.1)	1582.7 (624.1-3262.5)	p=0.01
Procalcitonin, ng/ml	0.35 (0.26-0.64)	0.52 (0.52-0.52)	p>0.1
Ferritin, ng/ml	1306 (777.9-1902.0)	no data	-
ALAT U/l	20.5 (12-30)	17 (16-19)	p>0.1
pO2, mmHg	71.7 (54.2-87.6)	71 (60.1-73.9)	p>0.1
pCO2, mmHg	33.85 (28.6-36.9)	27.2 (24.9-35.5)	p>0.1

Data is n (%) or median (IQR), unless otherwise specified; HD, hemodialyzed; ACE, angiotensin converting enzyme; ARB, angiotensin II receptor blocker; LMWH, low molecular weight heparin.
